# Associations of Glycemic Control With Cardiovascular Outcomes Among US Hemodialysis Patients With Diabetes Mellitus

**DOI:** 10.1161/JAHA.117.005581

**Published:** 2017-06-07

**Authors:** Jinnie J. Rhee, Yuanchao Zheng, Maria E. Montez‐Rath, Tara I. Chang, Wolfgang C. Winkelmayer

**Affiliations:** ^1^ Division of Nephrology Department of Medicine Stanford University School of Medicine Palo Alto CA; ^2^ Section of Nephrology Department of Medicine Selzman Institute for Kidney Heath Baylor College of Medicine Houston TX

**Keywords:** cardiovascular outcomes, diabetes mellitus, glycemic control, hemodialysis, Cardiovascular Disease, Diabetes, Type 1, Diabetes, Type 2, Epidemiology, Nephrology and Kidney

## Abstract

**Background:**

There is a lack of data on the relationship between glycemic control and cardiovascular end points in hemodialysis patients with diabetes mellitus.

**Methods and Results:**

We included adult Medicare‐insured patients with diabetes mellitus who initiated in‐center hemodialysis treatment from 2006 to 2008 and survived for >90 days. Quarterly mean time‐averaged glycated hemoglobin (HbA_1c_) values were categorized into <48 mmol/mol (<6.5%) (reference), 48 to <58 mmol/mol (6.5% to <7.5%), 58 to <69 mmol/mol (7.5% to <8.5%), and ≥69 mmol/mol (≥8.5%). Medicare claims were used to identify outcomes of cardiovascular mortality, nonfatal myocardial infarction (MI), fatal or nonfatal MI, stroke, and peripheral arterial disease. We used Cox models as a function of time‐varying exposure to estimate multivariable adjusted hazard ratios and 95%CI for the associations between HbA_1c_ and time to study outcomes in a cohort of 16 387 eligible patients. Patients with HbA_1c_ 58 to <69 mmol/mol (7.5% to <8.5%) and ≥69 mmol/mol (≥8.5%) had 16% (CI, 2%, 32%) and 18% (CI, 1%, 37%) higher rates of cardiovascular mortality (*P*‐trend=0.01) and 16% (CI, 1%, 33%) and 15% (CI, 1%, 32%) higher rates of nonfatal MI (*P*‐trend=0.05), respectively, compared with those in the reference group. Patients with HbA_1c_ ≥69 mmol/mol (≥8.5%) had a 20% (CI, 2%, 41%) higher rate of fatal or nonfatal MI (*P*‐trend=0.02), compared with those in the reference group. HbA_1c_ was not associated with stroke, peripheral arterial disease, or all‐cause mortality.

**Conclusions:**

Higher HbA_1c_ levels were significantly associated with higher rates of cardiovascular mortality and MI but not with stroke, peripheral arterial disease, or all‐cause mortality in this large cohort of hemodialysis patients with diabetes mellitus.

**Clinical Perspective**

**What Is New?**

- Taking advantage of 2 unusually large and detailed data sources as well as advanced methodology such as multiple imputation and time‐varying extended Cox models, the present study demonstrates that higher HbA_1c_ levels are significantly associated with higher rates of cardiovascular mortality and MI.

**What Are the Clinical Implications?**

- Although further research is needed to establish causal relationships between glycemic control and cardiovascular complications in patients on hemodialysis with DM, healthcare professionals should be aware of the cardiovascular disease risk associated with poor glycemic control in this patient population.

## Introduction

Diabetes mellitus (DM) is the leading cause of end‐stage renal disease (ESRD), reported in 45% of patients initiating treatment for ESRD in 2011.^1^ DM is also a major risk factor for cardiovascular disease, and cardiovascular disease accounted for 41% of deaths in US patients with ESRD in 2011.^2^, ^3^, ^4^ Previous studies have shown that patients on hemodialysis with DM have higher rates of several comorbidities and experience poorer clinical outcomes compared with patients without DM. Randomized trials have demonstrated that DM management or tight glycemic control through monitoring of blood glucose and glycated hemoglobin A_1c_ (HbA_1c_) levels can slow the progression of diabetic nephropathy^5^, ^6^ and reduce the risk of cardiovascular complications including myocardial infarction (MI) and coronary artery disease.^2^ Although these trials excluded patients on hemodialysis, it has been argued that better glycemic control is key toward preventing DM‐related complications in this population.^3^, ^7^, ^8^, ^9^ In fact, surprisingly few studies have investigated the association between HbA_1c_ and clinical outcomes in the dialysis population.^3^, ^10^, ^11^, ^12^, ^13^, ^14^, ^15^, ^16^ Most of these studies focused only on all‐cause mortality as the outcome and were limited by small sample size and correspondingly low statistical power.^11^, ^12^, ^13^, ^15^

Furthermore, there is evidence that non‐Hispanic blacks, Hispanics, and Asians have a lower risk of developing cardiovascular complications of DM compared with non‐Hispanic whites, whereas mortality from cardiovascular complications is disproportionately higher in minorities.^17^, ^18^, ^19^ However, whether race or ethnicity modifies the associations of glycemic control with cardiovascular outcomes in US patients on dialysis remains unclear.

Given these evidence gaps, in this study we examined the association between glycemic control and cardiovascular outcomes in a large cohort of US patients on hemodialysis with DM and assessed for effect modification by race and ethnicity.

## Methods

### Data Source

We used data from the US Renal Data System (USRDS), the national registry for patients with ESRD,^1^ and data from the electronic health records (EHR) of DaVita, Inc, the second largest dialysis provider in the United States. We merged information covering years 2006 to 2011 from both sources using a crosswalk of anonymized patient identifiers generated by the USRDS Coordinating Center, with approval by the Centers for Medicare and Medicaid Services and the National Institutes of Diabetes and Digestive and Kidney Disease. The USRDS contains demographic and certain comorbidity data for almost all US patients with ESRD as well as data from final‐action Medicare claims (Parts A, B, D) for eligible patients, and reported cause of death from the ESRD Death Notification (form CMS‐2746). The DaVita EHR provides highly granular and longitudinal data on laboratory values, including HbA_1c_, all measured in a central laboratory, as well as on vital signs and hemodialysis‐related parameters measured at the point of care.

### Study Population

**Figure 1:**
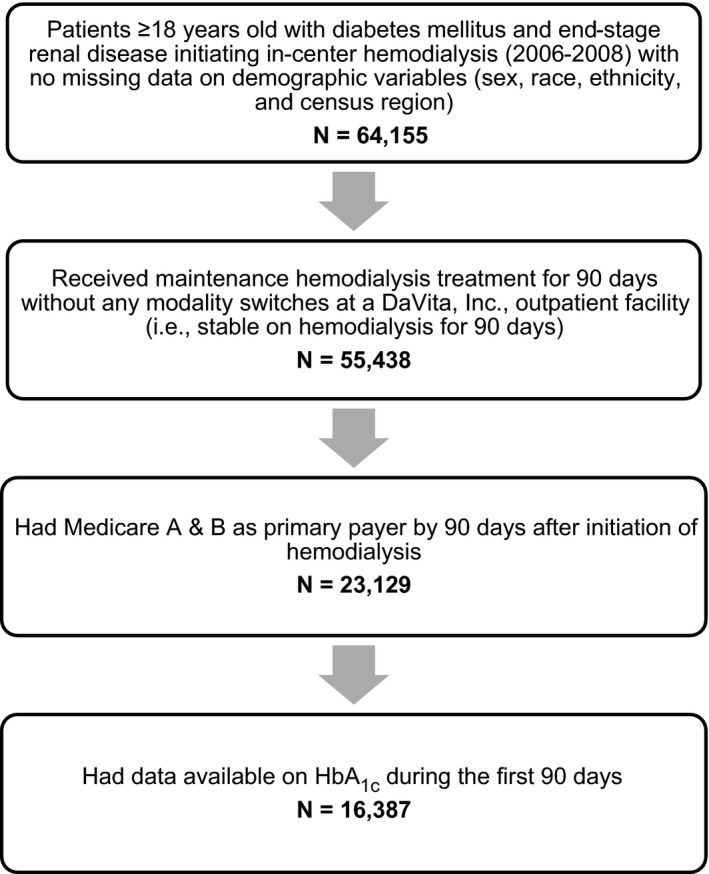
Study population derived from the US Renal Data System and electronic health records of DaVita, Inc.

The study population included all adult patients (≥18 years old) with incident ESRD between 2006 and 2008 and with DM reported as a comorbidity or cause of kidney disease in the ESRD Medical Evidence Report (form CMS‐2728) with no missing data on sex, race, ethnicity, and census region (N=64 155) (Figure 1). We restricted the cohort to those who received maintenance hemodialysis treatment at a DaVita outpatient facility and did not change to a different type of ESRD treatment (eg, peritoneal dialysis or kidney transplant) for 90 days (N=55 438). Because we relied on Medicare payment claims information to ascertain comorbid conditions and outcomes (see below), we further restricted the cohort to patients with Medicare fee‐for‐service (Parts A+B) as their primary payer by 90 days after initiation of hemodialysis (N=23 129). US Medicare insures all qualifying individuals starting at age 65 years and is mandated to insure all eligible patients with ESRD regardless of their age. Specifically, Medicare covers younger patients on the first day of the fourth month of ESRD, which for the majority of patients is between day 61 and 90 after incident ESRD (first treatment date). Hence, our cohort selection criteria required Medicare eligibility at day 90 to reflect near‐universal healthcare coverage for all patients with ESRD regardless of age and race/ethnicity. The only relevant group of younger patients who do not qualify for Medicare are those who receive health insurance through their employer (those have to wait additional 30 months for Medicare eligibility), but this is quite uncommon, as most patients are not in the workforce when they reach ESRD. We further excluded 6742 patients who did not have any data on HbA_1c_ during the first 90 days. The final cohort consisted of 16 387 patients. In the analysis of peripheral arterial disease (PAD), we additionally excluded 91 patients with preexisting PAD because PAD is often not an acute clinical condition. This study was approved by an institutional review board of Stanford University and conducted in accordance with the Declaration of Helsinki guidelines. Due to the unidentified nature of the data, patient consent was not deemed necessary and was not required.

### Primary Exposure

We abstracted HbA_1c_ data from the DaVita EHR. We divided the time since hemodialysis initiation into 90‐day quarters and averaged all available HbA_1C_ values within that quarter. This time‐varying quarterly mean HbA_1c_ level was our primary exposure. HbA_1c_ values were categorized into 4 groups: (1) <48 mmol/mol (<6.5%) (reference); (2) 48 to <58 mmol/mol (6.5% to <7.5%); (3) 58 to <69 mmol/mol (7.5% to <8.5%); and (4) ≥69 mmol/mol (≥8.5%).

### Primary Outcomes

We identified all‐cause mortality and the following cardiovascular outcomes using Medicare billing claims and the Death Notification form: cardiovascular mortality, nonfatal MI, fatal or nonfatal MI, fatal or nonfatal ischemic or hemorrhagic stroke, and PAD. With the exception of PAD, our group has previously established algorithms to identify these cardiovascular events from Medicare claims.^20^, ^21^ PAD was defined using previously established diagnosis codes and diagnosis‐related groups (Table 1).^22^

**Table 1:** Diagnosis Codes and Diagnosis‐Related Groups Used to Define PAD^22^

Must have 1 of the following diagnosis codes	
440.0	Atherosclerosis of aorta
440.20	Atherosclerosis of native arteries of extremities, unspecified
440.21	Atherosclerosis of native arteries of extremities, with intermittent claudication
440.22	Atherosclerosis of native arteries of extremities, with rest pain
440.23	Atherosclerosis of native arteries of extremities, with ulceration
440.24	Atherosclerosis of native arteries of extremities, with gangrene
440.31	Atherosclerosis of autologous vein bypass graft of extremities
440.9	Atherosclerosis nonspecific
442.3	Lower extremity aneurysm
443.9	Peripheral vascular disease, unspecified
444.2X	Arterial embolism and thrombosis of arteries of the extremities
444.81	Arterial embolism and thrombosis of other specified artery, iliac artery
AND be assigned to 1 of the following DRGs	
5	Extracranial vascular procedures
110	Major cardiovascular procedures with CC
111	Major cardiovascular procedures without CC
113	Amputation for circulatory system disorders except upper limb and toe
114	Upper limb and toe amputation for circulatory system disorders
124	Circulatory disorders except acute myocardial infection
130	Peripheral vascular disorders with CC
131	Peripheral vascular disorders without CC
132	Atherosclerosis with CC
133	Atherosclerosis without CC
213	Amputation for musculoskeletal system and connective tissue disorders
271	Skin ulcers
285	Amputation of lower limb for endocrine, nutritional, and metabolic disorders
287	Skin grafts and wound debridement for endocrine, nutritional, and metabolic disorders
478	Other vascular procedures with CC
479	Other vascular procedures without CC

CC indicates complications and comorbidities; DRG, diagnosis‐related group.

### Covariates

Information on age, sex, reported race (white, black, Asian, Native American, Pacific Islander, and other) and Hispanic ethnicity, and reported comorbidities were obtained from the Medical Evidence Report (form CMS‐2728) in the USRDS. Comorbidities were also obtained from claims using the International Classification of Diseases, Ninth Revision, diagnosis and procedure codes from at least 1 inpatient or 2 or more outpatient encounters separated by at least 1 day. We combined information from both the Medical Evidence Report and claims data to define baseline comorbidities and used claims thereafter to create time‐updated comorbidities in increments of 90‐day quarters. No individual‐level socioeconomic data were available in USRDS or the EHRs. As a result, we obtained area‐level socioeconomic data from the US Census Bureau American Community Survey. We were constrained to use ZIP code as the area of analysis in order to match the smallest indicator of geography available in the registry data. Although for some outcomes, ZIP code can lead to unstable associations due to heterogeneity of characteristics within a ZIP code, for most outcomes examined, results have been shown to be consistent with smaller census‐defined levels of geography.^23^ Neighborhood‐level socioeconomic variables at the ZIP‐code level included median rent, median household income, percentage living below the federal poverty line, percentage unemployed among those 16 years or older, and percentage with less than a high‐school education among those 25 years or older. Laboratory values, vital signs, and derived biometric parameters (normalized protein catabolic rate), and body mass index (BMI) were abstracted from the EHR. Sociodemographic variables, estimated glomerular filtration rate (eGFR), and BMI were ascertained at time of hemodialysis initiation only. All available laboratory variables and blood pressures were averaged within the 90‐day quarters and treated as time‐varying variables defined in the quarter preceding outcome ascertainment. All laboratory parameters including HbA_1c_ were measured using a single assay. HbA_1c_ was measured directly in the whole‐blood sample using high‐performance liquid chromatography and the Tosoh Bioscience (Tokyo, Japan) platforms known as G7 and G8. The methods were National Glycohemoglobin Standardization Program and International Federation of Clinical Chemistry certified.

We conducted a sensitivity analysis to examine the extent to which each covariate had an influence on the final model for each outcome of interest. We removed the covariates one at a time from the full model and computed percentage change in the β estimates to determine whether the variable was a significant confounder according to the “10% rule” (Table S1).

### Statistical Analysis

We compared patients’ baseline characteristics across 4 different levels of baseline HbA_1c_ using counts and proportions for categorical variables and mean (standard deviation) or median (interquartile ranges) for continuous variables. We tested for linear trend across HbA_1c_ categories using the Cochran‐Armitage test for categorical variables and the mean t test with contrasts for continuous variables.

We defined the index date as 90 days after hemodialysis initiation and followed the patients from the index date until an event of interest occurred. We censored at the end of the study period (December 31, 2011) or when patients stopped in‐center hemodialysis treatment or were lost to follow‐up, lost Medicare Parts A & B coverage, or died from any cause.

For each outcome, we calculated unadjusted incidence rates, defined as the number of events over person‐time observed, across the 4 baseline HbA_1c_ categories. We applied a Cox model as a function of time‐varying exposure (extended Cox) to the HbA_1c_ level categories to compute adjusted hazard ratios for each outcome, with the lowest category of mean HbA_1c_ level (<6.5%) as the referent. Hazard ratios were adjusted in 4 nested models: model 1, adjusted for year of incident ESRD; model 2, additionally adjusted for census division, sociodemographic variables, and Medicare/Medicaid dual eligibility; model 3, additionally adjusted for baseline BMI, eGFR, and time‐updated comorbidities; and model 4 additionally adjusted for time‐varying laboratory variables. We included in the corresponding model quadratic terms for all laboratory variables, BMI, and eGFR to account for the nonlinearity of the data. Standard errors were robustly estimated using sandwich estimators. We tested the linear effect of the exposure using contrast and tested for effect modification by race (white, black) and ethnicity (Hispanic, non‐Hispanic) by including a multiplicative interaction term in the model. Given the small number of black Hispanic patients (N=49), these patients were not included in the analysis.

#### Missing Data

Missing data were handled using multiple imputation methods with a fully conditional specification approach as implemented in Stata, and 5 imputed data sets were obtained for each outcome.^24^, ^25^ For the outcomes of cardiovascular and all‐cause mortality, 45 438 out of 161 155 records (28.2%) were incomplete. At the patient level this corresponded to 1915 patients having at least 1 variable missing for every quarter the patient was kept in the analysis. The percentage of missing values across all the variables varied between 0% and 19.5%. For time‐invariant variables, eGFR, BMI, and socioeconomic variables had missing values with the largest amount of missing data coming from median rent with 3.1%. We required patients to have baseline exposure but allowed follow‐up values to be missing. Forty‐eight percent of patients had at least 1 HbA_1c_ record missing with the number of HbA_1c_ records missing ranging between 4.2% and 95.7%. Laboratory measurements were also allowed to vary over time, and these could have missing values for all records including baseline. For these, the percentage of records with missing values varied between 8.1% for variables predialysis: weight, mean arterial pressure and pulse pressure, and 19.5% for HbA_1c_. These statistics were similar for the other outcomes of interest.

Under multiple imputation, we assumed that the data were missing at random, conditional on observed variables. This is a reasonable assumption given the baseline and intermittent values of the missing variables. In addition to the exposure and all covariates included in the analysis model, the imputation model also included the event indicator and the Nelson‐Aalen estimator of the cumulative marginal hazard H(T), where T is the time to event or censoring.^26^ Given that patients are more likely to be missing time‐varying observations if they spend time in the hospital, we added as an auxiliary variable the total number of days the patient spent in the hospital in the same quarter when the exposure and other covariates were measured. The inclusion of auxiliary variables has been shown to improve the imputation model.^27^ Imputations were repeated by race/ethnicity to allow testing for effect modification. All statistical analyses were performed using SAS, version 9.4 (SAS Institute, Inc, Cary, NC) and Stata version 13.1 (Stata Statistical Software:Release 13; StataCorp, College Station, TX).

## Results

We identified 16 387 patients initiating hemodialysis with DM, with mean HbA_1c_ of 48 mmol/mol (6.5%) and mean age of 64.5 years at baseline (Table 2). Patients with higher baseline HbA_1c_ levels tended to be younger, and the proportions of nonwhites were larger at higher HbA_1c_ levels. The prevalence of patients living below poverty was lower in patients with lower HbA_1c_, but the prevalence of most reported comorbidities including heart failure and coronary artery disease was higher in patients with lower HbA_1c_ levels. The mean eGFR reported at initiation of dialysis was the highest in patients with HbA_1c_ ≥69 mmol/mol (≥8.5%). Sensitivity analysis showed that variables such as hemoglobin, predialysis weight, and age were significant confounders for all outcomes in addition to nutritional status markers (albumin and normalized protein catabolic rate) for cardiovascular mortality and coronary artery disease as a comorbidity for nonfatal MI as well as fatal or nonfatal MI. Almost all variables were significant confounders for all‐cause mortality.

**Table 2:** Baseline Characteristics of 16 387 US Adult Patients With Diabetes Mellitus Initiating Maintenance Hemodialysis at a DaVita Outpatient Facility^a^^,^ b

Patient Characteristics	HbA_1c_ in mmol/mol (%)					
	All Patients (N=16 387)	<48 (<6.5) (N=9430)	48 to <58 (6.5 to <7.5) (N=4113)	58 to <69 (7.5 to <8.5) (N=1747)	≥69 (≥8.5) (N=1097)	*P* Value^c^
HbA_1c_, %	6.5±1.2	5.7±0.5	6.9±0.3	7.9±0.3	9.5±1.0	<0.001
Demographics						
Age, y	64.5±13.1	66.4±12.4	64.2±12.9	60.4±13.5	56.3±13.6	<0.001
Male sex, N (%)	8788 (53.6)	5062 (53.7)	2221 (54.0)	927 (53.1)	578 (52.7)	0.71
Race, N (%)						
White	10 624 (64.8)	6178 (65.5)	2703 (65.7)	1105 (63.3)	638 (58.2)	0.001
Black	4784 (29.2)	2727 (28.9)	1150 (28.0)	527 (30.2)	380 (34.6)	0.048
Asian	482 (2.9)	286 (3.0)	123 (3.0)	43 (2.5)	30 (2.7)	0.33
Pacific Islander	102 (0.6)	47 (0.5)	34 (0.8)	15 (0.9)	6 (0.5)	0.04
Native American	360 (2.2)	170 (1.8)	96 (2.3)	51 (2.9)	43 (3.9)	<0.001
Other/Multiracial	35 (0.2)	22 (0.2)	7 (0.2)	6 (0.3)	0 (0)	0.46
Hispanic ethnicity, N (%)	2825 (17.2)	1496 (15.9)	756 (18.4)	352 (20.1)	221 (20.1)	<0.001
Medicare/Medicaid dual eligibility	7003 (42.7%)	3811 (40.4)	1761 (42.8)	853 (48.8)	578 (52.7)	<0.001
Socioeconomic variables						
Median rent (US $)	862.0±275.0	870.9±277.8	858.0±274.5	843.4±261.4	829.1±269.1	0.04
Missing, N (%)	505 (3.1)	291 (3.1)	111 (2.7)	72 (4.1)	31 (2.8)	
Median household income ($)	49 287±19 781	50 030±20 443	49 008±19 084	47 375±17 991	46 986±18 867	0.10
Missing, N (%)	415 (2.5)	240 (2.5)	95 (2.3)	56 (3.2)	24 (2.2)	
% living below poverty	17.9±10.2	17.5±10.2	17.8±10.0	18.9±10.5	19.4±10.4	0.004
Missing, N (%)	407 (2.5)	236 (2.5)	93 (2.3)	54 (3.1)	24 (2.2)	
% unemployed	10.3±4.8	10.3±4.8	10.3±4.7	10.7±5.0	10.7±5.0	0.13
Missing, N (%)	406 (2.5)	235 (2.5)	93 (2.3)	54 (3.1)	24 (2.2)	
% <high school education	19.2±11.7	18.7±11.5	19.3±11.7	20.6±12.4	20.6±11.9	0.11
Missing, N (%)	402 (2.5)	234 (2.5)	91 (2.2)	53 (3.0)	24 (2.2)	
BMI, kg/m^2^	30.0±7.7	29.6±7.6	30.4±7.7	30.9±7.9	30.6±8.2	0.35
Missing, N (%)	264 (1.6)	157 (1.7)	57 (1.4)	35 (2.0)	15 (1.4)	
Predialysis weight, kg	84.8±22.3	83.3±22.0	86.1±21.9	88.3±22.9	87.5±23.7	0.74
Missing, N (%)	16 (0.1)	11 (0.1)	2 (0.1)	2 (0.1)	1 (0.1)	
Systolic blood pressure (mm Hg), median (25th to 75th percentile)	150.1 (136.9‐163.7)	148.2 (135.0‐161.8)	150.3 (137.3‐163.7)	154.9 (141.7‐168.0)	157.7 (145.3‐170.8)	0.025
Missing, N (%)	14 (0.1)	10 (0.1)	2 (0.1)	1 (0.1)	1 (0.1)	
Diastolic blood pressure (mm Hg), median (25th to 75th percentile)	75.2 (67.6‐84.1)	74.0 (66.6‐82.5)	75.0 (67.7‐83.8)	78.2 (70.9‐87.4)	82.5 (74.0‐91.0)	<0.001
Missing, N (%)	14 (0.1)	10 (0.1)	2 (0.1)	1 (0.1)	1 (0.1)	
Mean arterial pressure (mm Hg), median (25th to 75th percentile)	100.3 (91.5‐110.1)	98.9 (90.3‐108.4)	100.1 (91.6‐110.0)	104.4 (95.3‐113.6)	107.6 (99.0‐117.4)	<0.001
Missing, N (%)	14 (0.1)	10 (0.1)	2 (0.1)	1 (0.1)	1 (0.1)	
Pulse pressure (mm Hg), median (25th to 75th percentile)	73.6 (64.3‐83.2)	73.1 (63.7‐82.7)	74.1 (65.1‐83.3)	75.3 (65.9‐84.5)	74.5 (65.0‐83.8)	0.39
Missing, N (%)	14 (0.1)	10 (0.1)	2 (0.1)	1 (0.1)	1 (0.1)	
Reported comorbidities, N (%)						
Heart failure	9292 (56.7)	5471 (58.0)	2340 (56.9)	940 (53.8)	541 (49.3)	<0.001
Arrhythmias	1151 (7.0)	743 (7.9)	272 (6.6)	91 (5.2)	45 (4.1)	<0.001
Coronary artery disease	6889 (42.0)	4010 (42.5)	1787 (43.4)	706 (40.4)	386 (35.2)	0.007
Other cardiac disease	2935 (17.9)	1789 (19.0)	729 (17.7)	260 (14.9)	157 (14.3)	<0.001
Peripheral arterial disease	4360 (26.6)	2541 (26.9)	1104 (26.8)	448 (25.6)	267 (24.3)	0.12
Hypertension	15 723 (95.9)	9048 (95.9)	3943 (95.9)	1682 (96.3)	1050 (95.7)	0.99
COPD	3564 (21.7)	2169 (23.0)	879 (21.4)	345 (19.7)	171 (15.6)	<0.001
Current tobacco use	1041 (6.4)	585 (6.2)	239 (5.8)	122 (7.0)	95 (8.7)	0.074
Cancer	1305 (8.0)	871 (9.2)	309 (7.5)	77 (4.4)	48 (4.4)	<0.001
Alcohol dependence	206 (1.3)	141 (1.5)	41 (1.0)	19 (1.1)	5 (0.5)	0.001
Laboratory measurements						
Platelet count (×10^3^/μL), median (25th to 75th percentile)	250.3 (200.9‐309.0)	243.0 (194.0‐300.3)	256.8 (206.7‐316.8)	264.0 (214.3‐325.7)	274.0 (223.8‐331.5)	0.01
Missing, N (%)	55 (0.3)	35 (0.4)	10 (0.2)	6 (0.3)	4 (0.4)	
White blood cell count, ×1000/mm^3^	8.0±2.6	7.9±2.5	8.2±2.7	8.3±2.4	8.3±2.4	0.83
Missing, N (%)	32 (0.2)	20 (0.2)	6 (0.1)	2 (0.1)	4 (0.4)	
Albumin, g/dL	3.4±0.5	3.4±0.5	3.5±0.4	3.4±0.4	3.3±0.4	<0.001
Missing, N (%)	13 (0.1)	9 (0.1)	3 (0.1)	0	1 (0.1)	
nPCR, g/(kg·d)	0.8±0.2	0.8±0.2	0.8±0.2	0.8±0.2	0.8±0.2	0.003
Missing, N (%)	2450 (15.0)	1374 (14.6)	674 (16.4)	256 (14.7)	146 (13.3)	
Ferritin, ng/mL	337.3±325.6	356.2±333.1	328.4±348.8	294.6±253.3	275.8±249.1	0.005
Missing, N (%)	120 (0.7)	72 (0.8)	31 (0.8)	10 (0.6)	7 (0.6)	
Estimated GFR, mL/(min·1.73 m^2^)	11.4±4.8	11.3±4.8	11.5±4.7	11.6±4.7	12.2±4.7	0.001
Missing, N (%)	256 (1.6)	152 (1.6)	57 (1.4)	30 (1.7)	17 (1.5)	
Hemoglobin, g/dL	11.6±1.2	11.6±1.2	11.7±1.2	11.8±1.1	11.7±1.1	0.20
Missing, N (%)	4 (0.1)	2 (0.1)	1 (0.1)	1 (0.1)	0	
Calcium, mg/dL	9.3±0.5	9.3±0.6	9.3±0.5	9.3±0.5	9.3±0.5	0.02
Missing, N (%)	15 (0.1)	7 (0.1)	7 (0.2)	1 (0.1)	0	
Phosphorus, mg/dL	5.0±1.2	5.0±1.2	5.0±1.1	5.1±1.1	5.1±1.1	0.02
Missing, N (%)	13 (0.1)	7 (0.1)	5 (0.1)	1 (0.1)	0	
PTH, pg/mL	371.2±283.7	368.6±298.9	363.4±256.0	382.0±268.0	405.1±269.8	<0.001
Missing, N (%)	78 (0.5)	46 (0.5)	19 (0.5)	9 (0.5)	4 (0.5)	

BMI indicates body mass index; COPD, chronic obstructive pulmonary disease; GFR, glomerular filtration rate; HbA_1c_, glycated hemoglobin; nPCR, normalized protein catabolic rate; PTH, parathyroid hormone.

a Reported as means and standard deviations unless noted otherwise.

b Variables are described using means and standard deviations for normally distributed continuous data, medians and 25th and 75th percentile values for nonnormally distributed data, and counts and proportions for categorical data.

c *P*‐values were computed using a 2‐sided trend analysis.

Total person time for cardiovascular mortality was 37 683 years during which 3355 events were reported for an unadjusted incidence rate of 8.9 per 100 person‐years. The unadjusted incidence rates (per 100 person‐years) for the other events of interest were 7.0 for nonfatal MI, 7.8 for fatal or nonfatal MI, 3.5 for stroke, and 0.7 for a PAD event, and 23.1 for all‐cause mortality. Patients in the highest HbA_1c_ category at baseline generally had the highest incidence rates for all cardiovascular outcomes of interest except PAD, whereas those in the lowest HbA_1c_ category had the highest incidence rate for all‐cause mortality (Table 3).

**Table 3:** Unadjusted Incidence Rates (per 100 Person‐Years) of Cardiovascular Outcomes According to HbA_1c_ Categories at Baseline

	HbA_1c_ in mmol/mol (%)															
	<48 (<6.5)				48 to <58 (6.5 to <7.5)				58 to <69 (7.5 to <8.5)				≥69 (≥8.5)			
	No. Events	Total PT (y)	Mean PT (y)	IR	No. Events	Total PT (y)	Mean PT (y)	IR	No. Events	Total PT (y)	Mean PT (y)	IR	No. Events	Total PT (y)	Mean PT (y)	IR
CV mortality	1883	21 482	2.3	8.8	842	9501	2.3	8.9	374	4134	2.4	9.0	256	2566	2.3	10.0
Hospitalized MI	1362	20 107	2.1	6.8	640	8783	2.1	7.3	268	3845	2.2	7.0	175	2351	2.1	7.4
Any MI	1525	20 107	2.1	7.6	727	8783	2.1	8.3	303	3845	2.2	7.9	199	2351	2.1	8.5
Any stroke	703	20 770	2.2	3.4	332	9179	2.2	3.6	129	3974	2.3	3.2	93	2454	2.2	3.8
PAD	151	21 181	2.3	0.7	73	9338	2.3	0.8	29	4076	2.3	0.7	13	2521	2.3	0.5
All‐cause mortality	5141	21 482	2.3	23.9	2166	9501	2.3	22.8	857	4134	2.4	20.7	545	2566	2.3	21.2

CV indicates cardiovascular; HbA_1c_, glycated hemoglobin; MI, myocardial infarction; PAD, peripheral arterial disease; PT, person‐time.

In model 1 (adjusted for year of incident ESRD), higher HbA_1c_ was associated with lower rate of cardiovascular mortality (*P*‐trend<0.001) (Table 4). When we additionally adjusted for census division, demographic variables such as age, sex, race/ethnicity, Medicare/Medicaid dual eligibility, and area‐level geocoded SES variables, this association was no longer significant; however, patients in the HbA_1c_ 48 to <58 mmol/mol (6.5% to <7.5%) category had a 16% (95% CI, 7%, 24%) lower rate of cardiovascular mortality compared with those with HbA_1c_ <48 mmol/mol (<6.5%). Further adjustment for baseline eGFR and BMI, and other time‐updated comorbidities did not lead to substantial changes in the observed associations, but additionally adjusting for time‐varying laboratory values changed the direction of the associations, and higher HbA_1c_ was now associated with higher rate of cardiovascular mortality (*P*‐trend=0.01). Patients with HbA_1c_ 58 to <69 mmol/mol (7.5% to <8.5%) and HbA_1c_ ≥69 mmol/mol (≥8.5%) had 16% (95% CI, 2%, 32%) and 18% (95% CI, 1%, 37%) higher rates of cardiovascular mortality, respectively, compared with those with HbA_1c_ <48 mmol/mol (<6.5%).

**Table 4:** Hazard Ratios (95% CI) of Cardiovascular Outcomes According to HbA_1c_ Categories

	No. Events	HbA_1c_ in mmol/mol (%)								*P* Trend
		<48 (<6.5)		48 to <58 (6.5 to <7.5)		58 to <69 (7.5 to <8.5)		≥69 (≥8.5)		
		HR	95% CI	HR	95% CI	HR	95% CI	HR	95% CI	
CV mortality	3355									
Model 1^a^		1.00	···	0.80	0.72, 0.88	0.79	0.70, 0.90	0.73	0.62, 0.85	<0.001
Model 2^b^		1.00	···	0.84	0.76, 0.93	0.91	0.80, 1.03	0.93	0.79, 1.09	0.57
Model 3^c^		1.00	···	0.85	0.77, 0.94	0.92	0.81, 1.05	0.92	0.78, 1.08	0.45
Model 4^d^		1.00	···	1.03	0.93, 1.15	1.16	1.02, 1.32	1.18	1.01, 1.37	0.01
Nonfatal MI	2445									
Model 1^a^		1.00	···	1.03	0.93, 1.14	1.03	0.90, 1.17	0.99	0.87, 1.13	0.89
Model 2^b^		1.00	···	1.07	0.97, 1.19	1.13	0.99, 1.29	1.19	1.04, 1.35	0.009
Model 3^c^		1.00	···	1.07	0.97, 1.19	1.13	0.99, 1.29	1.16	1.01, 1.33	0.02
Model 4^d^		1.00	···	1.10	0.99, 1.22	1.16	1.01, 1.33	1.15	1.01, 1.32	0.05
Fatal or nonfatal MI	2754									
Model 1^a^		1.00	···	0.99	0.90, 1.09	0.98	0.85, 1.12	1.00	0.87, 1.16	0.99
Model 2^b^		1.00	···	1.03	0.94, 1.13	1.08	0.94, 1.23	1.20	1.04, 1.39	0.01
Model 3^c^		1.00	···	1.03	0.94, 1.13	1.08	0.94, 1.23	1.18	1.02, 1.37	0.02
Model 4^d^		1.00	···	1.08	0.98, 1.19	1.13	0.98, 1.29	1.20	1.02, 1.41	0.02
Stroke	1257									
Model 1^a^		1.00	···	1.03	0.88, 1.21	0.88	0.71, 1.07	1.00	0.82, 1.22	0.65
Model 2^b^		1.00	···	1.07	0.91, 1.25	0.95	0.77, 1.16	1.14	0.93, 1.41	0.44
Model 3^c^		1.00	···	1.10	0.94, 1.28	0.98	0.80, 1.21	1.17	0.95, 1.44	0.32
Model 4^d^		1.00	···	1.13	0.96, 1.33	1.00	0.81, 1.23	1.13	0.92, 1.38	0.50
PAD	266									
Model 1^a^		1.00	···	0.95	0.70, 1.30	1.05	0.72, 1.53	0.88	0.57, 1.36	0.67
Model 2^b^		1.00	···	0.98	0.72, 1.33	1.12	0.77, 1.64	0.98	0.63, 1.51	0.93
Model 3^c^		1.00	···	0.97	0.71, 1.32	1.14	0.78, 1.66	0.94	0.61, 1.48	0.99
Model 4^d^		1.00	···	1.02	0.74, 1.39	1.21	0.82, 1.78	1.01	0.62, 1.64	0.80
All‐cause mortality	8709									
Model 1^a^		1.00	···	0.73	0.69, 0.77	0.64	0.59, 0.69	0.57	0.51, 0.63	<0.001
Model 2^b^		1.00	···	0.77	0.73, 0.81	0.74	0.68, 0.80	0.75	0.67, 0.83	<0.001
Model 3^c^		1.00	···	0.79	0.74, 0.83	0.77	0.71, 0.83	0.75	0.68, 0.83	<0.001
Model 4^d^		1.00	···	1.00	0.94, 1.06	1.01	0.93, 1.10	1.00	0.91, 1.10	0.96

CV indicates cardiovascular; ESRD, end‐stage renal disease; HbA_1c_, glycated hemoglobin; HR, hazard ratio; MI, myocardial infarction; PAD, peripheral arterial disease; SES, socioeconomic status.

a Model 1 adjusted for year of ESRD incidence.

b Model 2 adjust for year of ESRD incidence in addition to census division (a marker for location), demographic variables such as age, sex, race/ethnicity, Medicare/Medicaid dual eligibility, and area‐level geocoded SES variables such as median rent, median household income, percentage living below poverty, percentage unemployed, and percentage with less than high school education.

c Model 3 adjusted for variables in model 2, baseline body mass index, estimated glomerular filtration rate, and preexisting comorbidities including heart failure, arrhythmias, coronary artery disease, other cardiac disease, peripheral arterial disease, hypertension, chronic obstructive pulmonary disease, current tobacco use, cancer, and alcohol dependence.

d Model 4 adjusted for variables in model 3 and laboratory variables such as albumin, normalized protein catabolic rate, hemoglobin, platelet count, white blood cell count, ferritin, mean arterial pressure, pulse pressure, serum calcium, serum phosphorus, parathyroid hormone, and predialysis weight.

**Figure 2:**
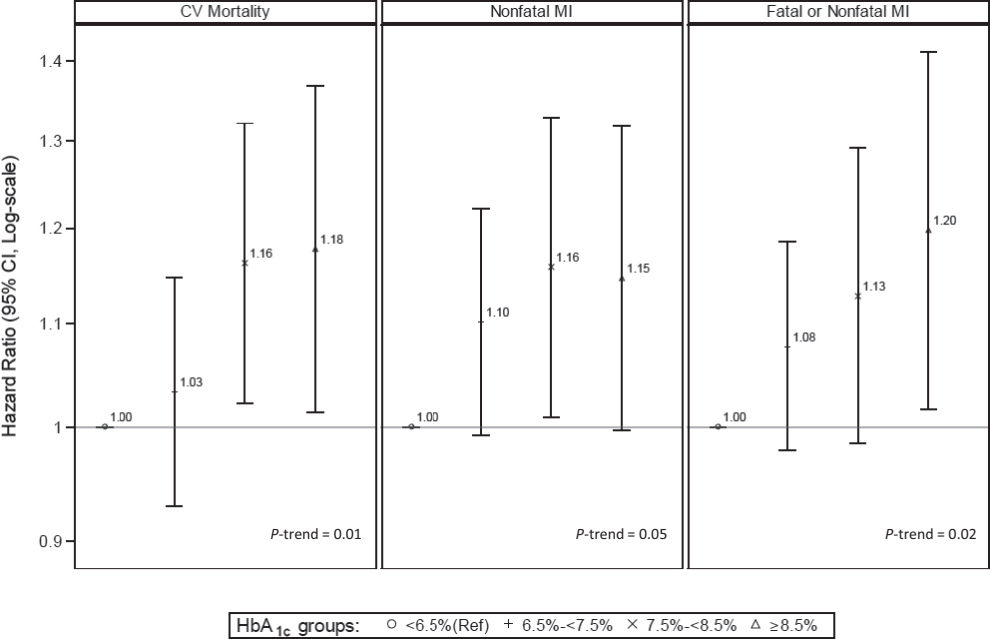
Hazard ratios, 95% CI, and *P*‐trends for the associations between time‐averaged glycated hemoglobin (HbA_1c_) and 3 different cardiovascular outcomes (cardiovascular mortality, nonfatal MI, and fatal or nonfatal MI) from model 4. This model adjusted for end‐stage renal disease incidence; census division (a marker for location); demographic variables such as age, sex, race/ethnicity; Medicare/Medicaid dual eligibility; area‐level geocoded socioeconomic standards variables such as median rent, median household income, percentage living below poverty, percentage unemployed, and percentage with less than high school education; baseline body mass index and estimated glomerular filtration rate; preexisting comorbidities including heart failure, arrhythmias, coronary artery disease, other cardiac disease, peripheral arterial disease, hypertension, chronic obstructive pulmonary disease, current tobacco use, cancer, and alcohol dependence; and laboratory variables such as albumin, normalized protein catabolic rate, hemoglobin, platelet count, white blood cell count, ferritin, mean arterial pressure, pulse pressure, serum calcium, serum phosphorus, parathyroid hormone level, and predialysis weight. For each outcome, HbA_1c_ categories are represented as the following: ᴏ, <6.5% (reference group); +, 6.5% to <7.5%; ×, 7.5% to <8.5%; and Δ, ≥8.5%. CV indicates cardiovascular; MI, myocardial infarction.

In models that only adjusted for year of ESRD incidence, census division, and sociodemographic variables, higher HbA_1c_ levels were also associated with higher rates of nonfatal MI (*P*‐trend=0.009) and fatal or nonfatal MI (*P*‐trend=0.01), with the rates being 19% (95% CI, 4%, 35%) and 20% (95% CI, 4%, 39%) higher, respectively, in patients with HbA_1c_ ≥69 mmol/mol (≥8.5%) compared with those with HbA_1c_ <48 mmol/mol (<6.5%). Additionally adjusting for baseline eGFR and BMI, and other time‐updated comorbidities did not largely change the magnitude of these associations. In the final model, which additionally adjusted for time‐varying laboratory variables, higher HbA_1c_ was associated with a higher rate of fatal or nonfatal MI (*P*‐trend=0.02), but the association with nonfatal MI was only marginally significant (*P*‐trend=0.05). There were 16% (95% CI, 1%, 33%) and 15% (95% CI, 1%, 32%) higher rates of nonfatal MI in patients with HbA_1c_ 58 to <69 mmol/mol (7.5% to <8.5%) and ≥69 mmol/mol (≥8.5%), respectively, compared with those with HbA_1c_ <48 mmol/mol (<6.5%). In patients with HbA_1c_ ≥69 mmol/mol (≥8.5%), there was a 20% (95% CI, 2%, 41%) higher rate of fatal or nonfatal MI compared with patients with HbA_1c_ <48 mmol/mol (<6.5%). The hazard ratios and 95% CI computed from the final model (model 4, which adjusted for all potential covariates) for the associations among time‐varying HbA_1c_ and cardiovascular mortality, nonfatal MI, and fatal or nonfatal MI are presented in Figure 2. There were no associations across HbA_1c_ categories with rates of any stroke or PAD in any of the 3 models (all *P*‐trend>0.05).

For all‐cause mortality, adjusting for year of ESRD incidence and subsequently for census division, sociodemographic variables, and comorbidities led to an inverse association with time‐averaged HbA_1c_, with rates being 21% (95% CI, 17%, 26%), 23% (95% CI, 17%, 29%), and 25% (95% CI, 17%, 32%) lower in patients with HbA_1c_ 6.5% to <7.5%, 7.5% to <8.5%, and ≥8.5%, respectively, compared with patients with HbA_1c_ <6.5%. However, this apparent association was no longer present after we adjusted for time‐varying laboratory variables (*P‐*trend=0.96).

There was no evidence of effect modification of the association between time‐averaged HbA_1c_ and any of the 5 cardiovascular outcomes of interest or all‐cause mortality by race and ethnicity (*P*‐for‐interaction >0.05 for all).

## Discussion

In this large prospective study of the association between glycemic control and cardiovascular outcomes in a cohort of incident US patients on hemodialysis with DM, we found that higher HbA_1c_ levels were significantly associated with higher rates of cardiovascular mortality and MI but not with the rates of stroke, PAD, or all‐cause mortality. We also found no evidence of effect modification of these associations by race and ethnicity. Our study had a relatively longer follow‐up than what has previously been reported and used a time‐varying analytic design with a comprehensive adjustment for various comorbidities and confounders including laboratory values.

In a 10‐year posttrial follow‐up study of the United Kingdom Prospective Diabetes Study, intensive glycemic treatment reduced MI and all‐cause mortality among patients with DM in the general population.^28^ In the ADVANCE and Veterans Affairs Diabetes Trial, however, tight glycemic control did not lead to an improvement in cardiovascular outcomes, whereas in the ACCORD trial, in which patients had underlying cardiovascular disease, intensive treatment was associated with higher cardiovascular mortality risk.^29^, ^30^, ^31^ Findings from these recent studies have collectively raised the question of whether glycemic control in populations with an underlying cardiovascular risk may do more harm than good, which is particularly applicable to patients on hemodialysis in whom the risks of cardiovascular morbidity and mortality are high.

Although there has been a lack of data on the relationship of HbA_1c_ with cardiovascular outcomes in hemodialysis patients with DM, several studies in the dialysis population have examined the association between HbA_1c_ and all‐cause mortality, and the literature is not unequivocal. Similar to our findings, a study by Williams et al showed no association between HbA_1c_ level and mortality after 1 year, but this study was limited by a single measure of HbA_1c_, short‐term follow‐up, and insufficient control of cardiovascular disease risk factors and confounders.^14^ When using time‐varying HbA_1c_ measures and adjusting for various confounders with follow‐up up to 3 years, Williams et al later found a U‐shaped association between HbA_1c_ and mortality risk, with the risks being higher at the extremes of HbA_1c_ (<48 mmol/mol [<6.5%] and >97 mmol/mol [>11%]).^32^ Similarly, in an analysis of data from the Dialysis Outcomes and Practice Patterns Study of patients on hemodialysis with DM, mortality was lowest at HbA_1c_ levels of 7% to 7.9% and increased at both lower and higher levels as HbA_1c_ moved further from 7% to 7.9%.^16^ This may be due to a combined effect of net catabolic balance and poor nutritional status of patients on hemodialysis as well as other clinical characteristics that are present in hemodialysis, all of which can have varying effects on glycemic control.^16^ Indeed, in our sensitivity analysis, we found that variables such as albumin, normalized protein catabolic rate, and predialysis weight in addition to age, hemoglobin, and mean arterial pressure were some of the strongest negative confounders that drove the association between glycemic control and cardiovascular mortality. This could explain why in our unadjusted analyses, we found an inverse association between glycemic control and cardiovascular mortality, but once we controlled for these aforementioned covariates, the risk associated with higher HbA_1c_ became more apparent in the fully adjusted model (model 4). Nonetheless, in contrast to these previous studies, we found no association with cardiovascular outcomes or all‐cause mortality at the lowest levels of HbA_1c_, regardless of whether we adjusted for nutritional factors and other covariates or not in fully adjusted models.

In another study of prevalent patients on hemodialysis with DM, there was an inverse association between time‐varying HbA_1c_ and mortality in unadjusted analyses. When adjusted for case‐mix and malnutrition‐inflammation markers and hemoglobin, the association shifted, with higher HbA_1c_ levels being incrementally associated with higher mortality risk.^3^ We also found similar associations so that higher HbA_1c_ appeared to be protective against all‐cause mortality in models that only adjusted for year of ESRD incidence, sociodemographic variables, and time‐updated comorbidities. Our study was methodologically different from some of these previous studies because we adjusted for a comprehensive set of time‐varying laboratory variables in the fully adjusted model over a longer follow‐up. In our fully adjusted model, we found that time‐varying HbA_1c_ was not associated all‐cause mortality. However, similar to the study by Kalantar‐Zadeh et al,^3^ our study also showed that the association between HbA_1c_ and cardiovascular mortality was sensitive to adjustment for laboratory and other biometric data. Our findings illustrate that relying only on billing data may sometimes yield spurious findings and that a combined data set of claims and clinical EHR data may be superior in asking certain clinical research questions in cohorts of hemodialysis.

Consistent with our findings, Drechsler et al^33^ did not find a significant association between baseline HbA_1c_ and the risk of stroke in the German Diabetes and Dialysis Study. However, higher HbA_1c_ was a strong risk factor for sudden death, whereas no association was found between baseline HbA_1c_ and risks of fatal or nonfatal MI and heart failure death.^33^ Although the effect of tight glycemic control on microvascular complications has been well established,^5^, ^28^, ^31^, ^34^ its effect on macrovascular complications remains controversial, and more research is warranted to further study these relationships in patients on hemodialysis with DM. In this study we observed only moderate associations between higher HbA_1c_ and increased rates of cardiovascular mortality and MI. Because ESRD patients constitute fewer than 5% of the chronic kidney disease patients in the United States, a majority of chronic kidney disease patients do not live long enough to develop ESRD or to start dialysis due to high rates of morbidity and mortality in these patients.^35^ Advanced kidney disease is also an independent risk factor for increased morbidity and mortality associated with atherosclerotic heart and cerebrovascular diseases.^36^ Therefore, patients who have survived to commence hemodialysis and be included in the analytic cohort of this study may have had the survival characteristics that placed them at an advantage, which could partially explain the lack of strikingly strong associations between higher HbA_1c_ and risks of cardiovascular outcomes.

Although there is some evidence of racial and ethnic disparities in complications of DM,^17^, ^18^, ^19^ we did not find any racial and ethnic differences in the incidence of any of the 5 cardiovascular outcomes or all‐cause mortality. Racial and ethnic disparities in DM complications can be explained by differences in access to health care, financial barriers to quality health care or health insurance, and comprehensive coverage.^18^ The lack of racial and ethnic disparities observed in our study may be attributable to relatively favorable selection of an insured population, such as that of Medicare beneficiaries with ESRD, in which financial barriers to health care are not as prominent, as they are seen regularly or at least monthly by a nephrologist.

We acknowledge the limitations of this study. First, these study findings may not be generalizable to other patient populations (eg, commercially insured patients), as the study was conducted in a selected cohort of patients on hemodialysis with DM who were insured by Medicare. Second, patients on hemodialysis may have falsely low HbA_1c_ levels due to shorter erythrocyte lifespan, lower erythrocyte concentration seen in anemia, or due to predominance of younger erythrocytes observed in patients who are on iron replacement therapy or erythropoiesis‐stimulating agents.^37^ Third, we could not determine and adjust for the severity of comorbidities because these data were abstracted from an administrative database. Fourth, we had relatively few PAD and stroke events, leading to wide confidence intervals and imprecise estimates, making it difficult to provide definitive conclusions with regard to the association between HbA_1c_ and these cardiovascular outcomes. Fifth, we used HbA_1c_ <48 mmol/mol (<6.5%) as the reference group, which also included patients with relatively low HbA_1c_ values. These patients have been shown to have particularly poor prognosis owing to cachexia and “burnt out” DM.^3^, ^16^ As a result, we cannot identify the best target HbA_1c_ for patients with DM and ESRD. Based on limited evidence, however, it is possible that higher HbA_1c_ goals rather than intensive glycemic control (HbA_1c_ <42 mmol/mol [<6.0%]) may be more appropriate in some patients with DM including those with chronic kidney disease.^16^, ^32^ Sixth, we excluded about one third of potentially eligible participants from the study because they were missing baseline HbA_1c_ data. These missing values were not imputed because multiple imputation methods are only appropriate for imputation of the exposure of interest if strong auxiliary variables are available to be used in the imputation model. In the absence of these variables, patients without baseline HbA_1c_ needed to be removed. Last, we cannot rule out residual confounding despite the adjustments made for potential confounding factors due to the observational nature of the study. Despite these limitations, the study herein takes advantage of 2 unusually large and detailed data sources to assess associations between glycemic control and various cardiovascular end points, adjusting for a wide array of demographic and socioeconomic factors as well as clinical parameters.

In conclusion, higher time‐varying HbA_1c_ was associated with increased rates of cardiovascular mortality and MI but not with rates of stroke, PAD, or all‐cause mortality. Further research, ideally in randomized outcomes trials, is needed to establish causal relationships between glycemic control and cardiovascular complications in patients on hemodialysis with DM.

## Sources of Funding

Rhee was supported by the National Institutes of Health‐National Institute for Diabetes and Digestive and Kidney Diseases grants T32 DK007357 and F32 DK103473. This study was conducted under data use agreements between Winkelmayer and the National Institute for Diabetes and Digestive and Kidney Diseases and DaVita Inc, respectively. Data reported herein were supplied by the USRDS. Interpretation and reporting of these data are the responsibility of the authors and in no way should be seen as official policy or interpretation of the US government. A National Institute for Diabetes and Digestive and Kidney Diseases officer reviewed the manuscript for compliance with federal research regulations and approved its submission for publication. Data acquisition was supported by grants R01 DK090181 and R01 DK095024.

## Disclosures

None.

## Supporting information


**Table S1.** One‐at‐a‐Time Sensitivity Analysis Examining the Extent to Which Each Covariate Has an Influence on the Final Model for Each Outcome of Interest

## References

[1] National Kidney Foundation . 2013 USRDS annual data report: atlas of chronic kidney disease and end‐stage renal disease in the United States. Am J Kidney Dis. 2014;63(supp):e1–e478.

[2] Gæde P , Vedel P , Larsen N , Jensen GV , Parving HH , Pedersen O . Multifactorial intervention and cardiovascular disease in patients with type 2 diabetes. N Engl J Med. 2003;348:383–393. 12556541 10.1056/NEJMoa021778

[3] Kalantar‐Zadeh K , Kopple JD , Regidor DL , Jing J , Shinaberger CS , Aronovitz J , McAllister CJ , Whellan D , Sharma K . A1C and survival in maintenance hemodialysis patients. Diabetes Care. 2007;30:1049–1055. 17337501 10.2337/dc06-2127

[4] United States Renal Data System: excerpts from the USRDS 2005 annual data report: atlas of end‐stage renal disease in the United States, National Institutes of Health, National Institutes of Diabetes and Digestive and Kidney Diseases. Am J Kidney Dis. 2006;47(suppl 1):1–286.16377379

[5] The Diabetes Control and Complications Trial Research Group .The effect of intensive treatment of diabetes on the development and progression of long‐term complications in insulin‐dependent diabetes mellitus. N Engl J Med. 1993;329:977–986. 8366922 10.1056/NEJM199309303291401

[6] Tanaka Y , Atsumi Y , Matsuoka K , Onuma T , Tohjima T , Kawamori R . Role of glycemic control and blood pressure in the development and progression of nephropathy in elderly Japanese NIDDM patients. Diabetes Care. 1998;21:116–120. 9538981 10.2337/diacare.21.1.116

[7] Abbott KC , Bakris GL . Treatment of the diabetic patient: focus on cardiovascular and renal risk reduction. Prog Brain Res. 2002;139:289–298. 12436944 10.1016/s0079-6123(02)39025-3

[8] Friedman EA . Renal syndromes in diabetes. Endocrinol Metab Clin North Am. 1996;25:293–324. 8799702 10.1016/s0889-8529(05)70326-1

[9] Kimmel PL , Varela MP , Peterson RA , Weihs KL , Simmens SJ , Alleyne S , Amarashinge A , Mishkin GJ , Cruz I , Veis JH . Interdialytic weight gain and survival in hemodialysis patients: effects of duration of ESRD and diabetes mellitus. Kidney Int. 2000;57:1141–1151. 10720966 10.1046/j.1523-1755.2000.00941.x

[10] Feldt‐Rasmussen B . Is there a need to optimize glycemic control in hemodialyzed diabetic patients? Kidney Int. 2006;70:1392–1394. 17024163 10.1038/sj.ki.5001886

[11] McMurray SD , Johnson G , Davis S , McDougall K . Diabetes education and care management significantly improve patient outcomes in the dialysis unit. Am J Kidney Dis. 2002;40:566–575. 12200809 10.1053/ajkd.2002.34915

[12] Morioka T , Emoto M , Tabata T , Shoji T , Tahara H , Kishimoto H , Ishimura E , Nishizawa Y . Glycemic control is a predictor of survival for diabetic patients on hemodialysis. Diabetes Care. 2001;24:909–913. 11347753 10.2337/diacare.24.5.909

[13] Oomichi T , Emoto M , Tabata T , Morioka T , Tsujimoto Y , Tahara H , Shoji T , Nishizawa Y . Impact of glycemic control on survival of diabetic patients on chronic regular hemodialysis: a 7‐year observational study. Diabetes Care. 2006;29:1496–1500. 16801568 10.2337/dc05-1887

[14] Williams M , Lacson E , Teng M , Ofsthun N , Lazarus J . Hemodialyzed type I and type II diabetic patients in the US: characteristics, glycemic control, and survival. Kidney Int. 2006;70:1503–1509. 16941022 10.1038/sj.ki.5001789

[15] Wu MS , Yu CC , Yang CW , Wu CH , Haung JY , Hong JJ , Chiang CF , Huang CC , Leu ML . Poor pre‐dialysis glycaemic control is a predictor of mortality in type II diabetic patients on maintenance haemodialysis. Nephrol Dial Transplant. 1997;12:2105–2110. 9351073 10.1093/ndt/12.10.2105

[16] Ramirez SP , McCullough KP , Thumma JR , Nelson RG , Morgenstern H , Gillespie BW , Inaba M , Jacobson SH , Vanholder R , Pisoni RL , Port FK , Robinson BM . Hemoglobin A(1c) levels and mortality in the diabetic hemodialysis population: findings from the Dialysis Outcomes and Practice Patterns Study (DOPPS). Diabetes Care. 2012;35:2527–2532. 22912431 10.2337/dc12-0573 PMC3507600

[17] Golden SH , Brown A , Cauley JA , Chin MH , Gary‐Webb TL , Kim C , Sosa JA , Sumner AE , Anton B . Health disparities in endocrine disorders: biological, clinical, and nonclinical factors—an Endocrine Society scientific statement. J Clin Endocrinol Metab. 2012;97:E1579–E1639. 22730516 10.1210/jc.2012-2043 PMC3431576

[18] Karter AJ , Ferrara A , Liu JY , Moffet HH , Ackerson LM , Selby JV . Ethnic disparities in diabetic complications in an insured population. JAMA. 2002;287:2519–2527. 12020332 10.1001/jama.287.19.2519

[19] Spanakis EK , Golden SH . Race/ethnic difference in diabetes and diabetic complications. Curr Diab Rep. 2013;13:814–823. 24037313 10.1007/s11892-013-0421-9 PMC3830901

[20] Lenihan CR , Montez‐Rath ME , Scandling JD , Turakhia MP , Winkelmayer WC . Outcomes after kidney transplantation of patients previously diagnosed with atrial fibrillation. Am J Transplant. 2013;13:1566–1575. 23721555 10.1111/ajt.12197 PMC3670777

[21] Nair SS , Mitani AA , Goldstein BA , Chertow GM , Lowenberg DW , Winkelmayer WC . Temporal trends in the incidence, treatment, and outcomes of hip fracture in older patients initiating dialysis in the United States. Clin J Am Soc Nephrol. 2013;8:1336–1342. 23660182 10.2215/CJN.10901012 PMC3731911

[22] Hirsch AT , Hartman L , Town RJ , Virnig BA . National health care costs of peripheral arterial disease in the Medicare population. Vasc Med. 2008;13:209–215. 18687757 10.1177/1358863X08089277

[23] Krieger N , Waterman P , Chen JT , Soobader M‐J , Subramanian S , Carson R . Zip code caveat: bias due to spatiotemporal mismatches between zip codes and US census‐defined geographic areas—the Public Health Disparities Geocoding Project. Am J Public Health. 2002;92:1100–1102. 12084688 10.2105/ajph.92.7.1100 PMC1447194

[24] Van Buuren S , Boshuizen HC , Knook DL . Multiple imputation of missing blood pressure covariates in survival analysis. Stat Med. 1999;18:681–694. 10204197 10.1002/(sici)1097-0258(19990330)18:6<681::aid-sim71>3.0.co;2-r

[25] Van Buuren S , Brand JP , Groothuis‐Oudshoorn C , Rubin DB . Fully conditional specification in multivariate imputation. J Stat Comput Simul. 2006;76:1049–1064.

[26] White IR , Royston P . Imputing missing covariate values for the Cox model. Stat Med. 2009;28:1982–1998. 19452569 10.1002/sim.3618 PMC2998703

[27] Montez‐Rath ME , Winkelmayer WC , Desai M . Addressing missing data in clinical studies of kidney diseases. Clin J Am Soc Nephrol. 2014;9:1328–1335. 24509298 10.2215/CJN.10141013 PMC4078963

[28] Holman RR , Paul SK , Bethel MA , Matthews DR , Neil HAW . 10‐year follow‐up of intensive glucose control in type 2 diabetes. N Engl J Med. 2008;359:1577–1589. 18784090 10.1056/NEJMoa0806470

[29] Duckworth W , Abraira C , Moritz T , Reda D , Emanuele N , Reaven PD , Zieve FJ , Marks J , Davis SN , Hayward R . Glucose control and vascular complications in veterans with type 2 diabetes. N Engl J Med. 2009;360:129–139. 19092145 10.1056/NEJMoa0808431

[30] Ismail‐Beigi F , Craven T , Banerji MA , Basile J , Calles J , Cohen RM , Cuddihy R , Cushman WC , Genuth S , Grimm RH . Effect of intensive treatment of hyperglycaemia on microvascular outcomes in type 2 diabetes: an analysis of the ACCORD randomised trial. Lancet. 2010;376:419–430. 20594588 10.1016/S0140-6736(10)60576-4 PMC4123233

[31] The ADVANCE Collaborative Group . Intensive blood glucose control and vascular outcomes in patients with type 2 diabetes. N Engl J Med. 2008;358:2560–2572. 18539916 10.1056/NEJMoa0802987

[32] Williams ME , Lacson E , Wang W , Lazarus JM , Hakim R . Glycemic control and extended hemodialysis survival in patients with diabetes mellitus: comparative results of traditional and time‐dependent Cox model analyses. Clin J Am Soc Nephrol. 2010;5:1595–1601. 20671217 10.2215/CJN.09301209 PMC2974399

[33] Drechsler C , Krane V , Ritz E , März W , Wanner C . Glycemic control and cardiovascular events in diabetic hemodialysis patients. Circulation. 2009;120:2421–2428. 19948978 10.1161/CIRCULATIONAHA.109.857268

[34] Stratton IM , Adler AI , Neil HAW , Matthews DR , Manley SE , Cull CA , Hadden D , Turner RC , Holman RR . Association of glycaemia with macrovascular and microvascular complications of type 2 diabetes (UKPDS 35): prospective observational study. BMJ. 2000;321:405–412. 10938048 10.1136/bmj.321.7258.405 PMC27454

[35] Kalantar‐Zadeh K , Block G , Humphreys MH , Kopple JD . Reverse epidemiology of cardiovascular risk factors in maintenance dialysis patients. Kidney Int. 2003;63:793–808. 12631061 10.1046/j.1523-1755.2003.00803.x

[36] Mann JF , Gerstein HC , Pogue J , Bosch J , Yusuf S . Renal insufficiency as a predictor of cardiovascular outcomes and the impact of ramipril: the HOPE randomized trial. Ann Intern Med. 2001;134:629–636. 11304102 10.7326/0003-4819-134-8-200104170-00007

[37] Rhee JJ , Ding VY , Rehkopf DH , Arce CM , Winkelmayer WC . Correlates of poor glycemic control among patients with diabetes initiating hemodialysis for end‐stage renal disease. BMC Nephrol. 2015;16:1. 10.1186/s12882-015-0204-4 PMC4673753 26645204

