# Supplementary material for: Associations of Glycemic Control With Cardiovascular Outcomes Among US Hemodialysis Patients With Diabetes Mellitus
Source: J Am Heart Assoc. 2017 Jun 7;6(6):e005581. doi: 10.1161/JAHA.117.005581 (PMC5669174; doi:10.1161/JAHA.117.005581)
Supplement: Supplementary file 1 — Table S1. One‐at‐a‐Time Sensitivity Analysis Examining the Extent to Which Each Covariate Has an Influence on the Final Model for Each Outcome of Interest [file JAH3-6-e005581-s001.pdf]

SUPPLEMENTAL MATERIAL

Table S1. One-at-a-time sensitivity analysis examining the extent to which each covariate has an influence on the final model for each outcome of interest

In this analysis, we removed the covariates one at a time from the full model and computed Δ as shown below to determine whether the variable was a significant confounder according to the 10% rule. The tables hereby present beta estimates and 95% CI across HbA1c categories for the full model as well as beta estimates and % change in these estimates for the reduced model after each covariate was removed one-by-one from the full model. We present data for all outcomes of interest.

$$\text{Full model: } \log H = \beta_{11}(\text{HbA1c2}) + \beta_{21}(\text{HbA1c3}) + \beta_{31}(\text{HbA1c4}) + \gamma_1 Z + \delta_1 X$$

$$\text{Reduced model: } \log H + \beta_{10}(\text{HbA1c2}) + \beta_{20}(\text{HbA1c3}) + \beta_{30}(\text{HbA1c4}) + \gamma_0 Z,$$

where covariate X was removed from the full model

$$\text{Compute } \Delta = \frac{\beta 10 - \beta 11}{\beta 10} \text{ x } 100\%$$

Confounder significant if $\Delta > 10\%$

Cardiovascular Mortality

	HbA1c2		HbA1c3		HbA1c4	
	β_{11}	95% CI	B_{21}	95% CI	B_{31}	95% CI
Full Model	0.032	(-0.073, 0.137)	0.150	(0.021, 0.279)	0.163	(0.013, 0.314)

		HbA1c2		HbA1c3		HbA1c4
**Variable Removed**	β_{10}	%change	β_{20}	%change	β_{30}	%change
Year of ESRD incidence	0.031	-1.842	0.149	-1.101	0.162	-1.02
Census division	0.028	-13.551	0.146	-2.752	0.156	-4.492
Age	0.021	-54.961	0.122	-23.483	0.102	-60.172
Sex	0.027	-16.628	0.144	-4.354	0.154	-5.869
Race	0.03	-7.79	0.145	-3.916	0.155	-5.127
Ethnicity	0.027	-17.778	0.144	-4.73	0.154	-5.848
Medicare/Medicaid dual eligibility	0.032	0.815	0.15	-0.014	0.164	0.27
Median rent	0.033	2.035	0.151	0.131	0.163	-0.243
Median household income	0.032	-0.978	0.15	-0.197	0.164	0.266
% living below poverty	0.032	-0.682	0.15	0.033	0.164	0.072
% unemployed	0.032	-0.816	0.15	-0.23	0.163	-0.231
% with less than high school education_	0.031	-1.725	0.15	-0.448	0.162	-0.691
BMI	0.032	1.325	0.15	-0.185	0.165	0.82
eGFR	0.038	15.758	0.158	4.823	0.174	6.296
Heart failure	0.032	0.815	0.15	-0.014	0.164	0.27
Arrhythmias	0.029	-12.112	0.146	-2.694	0.158	-3.52
Coronary artery disease	0.038	16.003	0.157	4.389	0.174	5.81
Other cardiac disease	0.03	-6.873	0.147	-2.59	0.159	-2.797
Peripheral arterial disease	0.036	10.201	0.153	1.616	0.168	2.499
Hypertension	0.032	-0.037	0.15	-0.048	0.163	-0.017
Chronic obstructive pulmonary disease	0.03	-5.264	0.145	-3.363	0.157	-4.408
Current tobacco use	0.032	0.106	0.15	0.014	0.163	-0.085
Cancer	0.032	0.845	0.151	0.263	0.164	0.15
Alcohol dependence	0.032	-0.663	0.15	-0.101	0.163	-0.254
Albumin	0.011	-190.307	0.143	-5.264	0.192	14.821
nPCR	0.017	-84.096	0.131	-14.341	0.144	-13.445
Hemoglobin	0.008	-319.358	0.115	-30.632	0.114	-43.474
Platelet count	0.025	-27.151	0.144	-4.699	0.157	-3.972
White blood cell count	0.036	11.125	0.16	5.9	0.171	4.144
Ferritin	0.033	3.243	0.152	0.827	0.167	2.002
Mean arterial pressure	0.022	-47.121	0.134	-11.791	0.131	-24.763
Pulse pressure	0.031	-3.254	0.143	-5.306	0.151	-7.97
Calcium	0.031	-3.327	0.148	-1.883	0.16	-2.412
Phosphorus	0.03	-7.692	0.148	-1.559	0.166	1.477
PTH	0.035	7.529	0.152	0.942	0.167	1.855
Predialysis weight	0.02	-61.568	0.132	-13.951	0.146	-11.632

Nonfatal MI

	HbA1c2		HbA1c3		HbA1c4	
	β_{11}	**95% CI**	B_{21}	**95% CI**	B_{31}	**95% CI**
Full Model	0.096	(-0.008, 0.200)	0.147	(0.009, 0.285)	0.137	(-0.003, 0.276)

**Variable Removed**	β_{10}	**HbA1c2 %change**	B_{20}	**HbA1c3 %change**	B_{30}	**HbA1c4 %change**
Year of ESRD incidence	0.096	0.024	0.148	0.462	0.137	0.516
Census division	0.098	2.278	0.15	2.38	0.141	3.094
Age	0.079	-21.178	0.105	-39.755	0.053	-157.567
Sex	0.095	-1.538	0.145	-1.634	0.133	-2.5
Race	0.095	-1.218	0.143	-3.06	0.13	-4.908
Ethnicity	0.092	-4.224	0.141	-4.376	0.13	-5.379
Medicare/Medicaid dual eligibility	0.095	-0.773	0.146	-0.528	0.135	-1.327
Median rent	0.096	-0.069	0.147	-0.005	0.137	0.091
Median household income	0.096	-0.136	0.147	-0.083	0.137	0.142
% living below poverty	0.096	0.038	0.147	0.018	0.137	-0.031
% unemployed	0.096	0.158	0.147	0.133	0.137	0.248
% with less than high school education_	0.097	0.886	0.148	0.877	0.138	1.149
BMI	0.096	0.04	0.147	0.018	0.137	0.17
eGFR	0.098	2.344	0.149	1.736	0.138	1.363
Heart failure	0.096	0.037	0.148	0.958	0.141	3.021
Arrhythmias	0.096	-0.309	0.146	-0.294	0.136	-0.517
Coronary artery disease	0.11	12.547	0.165	11.098	0.159	14.346
Other cardiac disease	0.092	-4.168	0.141	-4.262	0.129	-5.729
Peripheral arterial disease	0.099	2.801	0.149	1.73	0.142	3.472
Hypertension	0.096	0.084	0.147	0.274	0.137	-0.033
Chronic obstructive pulmonary disease	0.096	-0.305	0.146	-0.581	0.135	-0.887
Current tobacco use	0.096	-0.117	0.147	0.035	0.136	-0.409
Cancer	0.094	-1.839	0.144	-1.651	0.134	-1.645
Alcohol dependence	0.097	1.047	0.148	0.672	0.139	1.671
Albumin	0.097	1.288	0.154	4.802	0.159	13.825
nPCR	0.091	-5.847	0.14	-5.004	0.129	-6.238
Hemoglobin	0.083	-16.094	0.126	-16.334	0.11	-24.235
Platelet count	0.095	-1.317	0.145	-1.156	0.134	-1.762
White blood cell count	0.098	1.757	0.149	1.716	0.14	2.293
Ferritin	0.097	0.949	0.148	1.01	0.139	1.47
Mean arterial pressure	0.098	2.14	0.15	2.189	0.143	4.632
Pulse pressure	0.096	0.148	0.148	0.68	0.139	1.617
Calcium	0.094	-1.909	0.143	-2.442	0.133	-2.889
Phosphorus	0.093	-3.532	0.144	-2.114	0.139	1.871
PTH	0.098	1.775	0.147	0.116	0.138	0.928
Predialysis weight	0.083	-15.919	0.127	-15.368	0.117	-16.526

Fatal or Nonfatal MI

	HbA1c2		HbA1c3		HbA1c4	
	β_{11}	95% CI	B_{21}	95% CI	B_{31}	95% CI
Full Model	0.074	(-0.023, 0.170)	0.120	(-0.016, 0.256)	0.181	(0.016, 0.345)

		HbA1c2		HbA1c3		HbA1c4
Variable Removed	β_{10}	%change	B_{20}	%change	B_{30}	%change
Year of ESRD incidence	0.074	0.438	0.122	1.381	0.183	1.032
Census division	0.076	2.263	0.123	2.556	0.185	2.148
Age	0.058	-27.5	0.08	-50.449	0.099	-82.27
Sex	0.072	-2.564	0.117	-2.822	0.176	-2.565
Race	0.072	-2.195	0.115	-4.267	0.174	-4.027
Ethnicity	0.071	-4.385	0.115	-4.163	0.175	-3.055
Medicare/Medicaid dual eligibility	0.073	-0.697	0.12	-0.416	0.18	-0.641
Median rent	0.074	0.003	0.12	0.008	0.181	0.006
Median household income	0.074	-0.11	0.12	-0.044	0.181	0.141
% living below poverty	0.074	-0.056	0.12	-0.039	0.181	0.025
% unemployed	0.074	0.277	0.12	0.234	0.181	0.27
% with less than high school education_	0.074	0.441	0.12	0.374	0.181	0.351
BMI	0.074	0.205	0.12	0.088	0.181	0.388
eGFR	0.075	1.069	0.12	0.397	0.179	-0.657
Heart failure	0.074	0.393	0.121	0.943	0.185	2.374
Arrhythmias	0.074	-0.439	0.12	-0.345	0.18	-0.338
Coronary artery disease	0.087	15.233	0.137	12.671	0.201	10.191
Other cardiac disease	0.07	-5.746	0.114	-5.666	0.173	-4.621
Peripheral arterial disease	0.077	3.58	0.123	2.071	0.185	2.472
Hypertension	0.074	0.05	0.12	0.2	0.181	-0.022
Chronic obstructive pulmonary disease	0.073	-0.643	0.119	-1.079	0.179	-1.054
Current tobacco use	0.074	-0.182	0.12	0.016	0.18	-0.332
Cancer	0.072	-2.802	0.117	-2.405	0.178	-1.365
Alcohol dependence	0.075	1.439	0.121	1.02	0.183	1.418
Albumin	0.074	-0.458	0.127	5.263	0.204	11.588
nPCR	0.069	-7.457	0.113	-6.315	0.173	-4.524
Hemoglobin	0.059	-24.304	0.098	-22.912	0.152	-19.147
Platelet count	0.072	-1.996	0.118	-1.649	0.178	-1.489
White blood cell count	0.076	3.113	0.124	3.105	0.185	2.257
Ferritin	0.075	1.528	0.122	1.382	0.184	1.606
Mean arterial pressure	0.076	2.303	0.123	2.096	0.185	2.581
Pulse pressure	0.074	-0.031	0.12	-0.148	0.18	-0.286
Calcium	0.072	-2.607	0.116	-3.121	0.176	-2.773
Phosphorus	0.07	-5.357	0.117	-2.778	0.182	1.001
PTH	0.075	2.097	0.12	0.127	0.182	0.564
Predialysis weight	0.061	-20.08	0.101	-18.607	0.162	-11.554

Stroke

	HbA1c2		HbA1c3		HbA1c4	
	β_{11}	95% CI	B_{21}	95% CI	B_{31}	95% CI
Full Model	0.123	(-0.041, 0.287)	-0.001	(-0.206, 0.205)	0.119	(-0.084, 0.322)

Variable Removed	β_{10}	HbA1c2 %change	B_{20}	HbA1c3 %change	B_{30}	HbA1c4 %change
Year of ESRD incidence	0.123	-0.368	-0.003	82.597	0.117	-1.888
Census division	0.122	-0.747	-0.001	-0.19	0.12	0.552
Age	0.105	-17.422	-0.044	98.794	0.036	-233.695
Sex	0.125	1.861	0.003	118.436	0.124	4.049
Race	0.12	-2.575	-0.005	88.339	0.115	-3.907
Ethnicity	0.125	1.232	0.002	129.949	0.121	1.922
Medicare/Medicaid dual eligibility	0.124	0.368	0	-120.921	0.12	0.713
Median rent	0.123	0.07	0	-28.467	0.119	0.225
Median household income	0.123	0.085	0	-8.866	0.119	-0.325
% living below poverty	0.123	0.111	-0.001	-1.734	0.118	-0.699
% unemployed	0.123	0.204	0	-78.983	0.12	0.444
% with less than high school education_	0.123	-0.133	-0.001	28.358	0.119	-0.234
BMI	0.124	1.115	0.001	142.512	0.122	2.21
eGFR	0.122	-1.06	-0.002	76.883	0.117	-2.097
Heart failure	0.123	0.169	0	336.771	0.12	1.131
Arrhythmias	0.122	-0.616	-0.002	65.49	0.118	-1.069
Coronary artery disease	0.125	1.348	0.002	122.018	0.122	2.406
Other cardiac disease	0.113	-8.813	-0.019	97.275	0.096	-23.656
Peripheral arterial disease	0.124	0.469	0	-1747.9	0.12	1.133
Hypertension	0.123	0.037	0	-1253.15	0.119	-0.003
Chronic obstructive pulmonary disease	0.123	0.123	0	-633.221	0.12	0.602
Current tobacco use	0.123	0.011	0	-25.118	0.119	0.183
Cancer	0.124	0.701	0.001	164.785	0.12	1.014
Alcohol dependence	0.121	-2.004	-0.003	82.388	0.114	-4.177
Albumin	0.123	-0.414	0.012	104.297	0.163	27.022
nPCR	0.113	-8.533	-0.014	96.104	0.104	-14.044
Hemoglobin	0.112	-9.694	-0.017	96.874	0.097	-22.219
Platelet count	0.123	-0.405	-0.001	56.507	0.119	-0.341
White blood cell count	0.128	3.502	0.005	109.877	0.125	4.514
Ferritin	0.123	0.012	-0.001	34.491	0.119	-0.229
Mean arterial pressure	0.13	5.336	0.011	104.789	0.144	17.209
Pulse pressure	0.123	-0.128	-0.002	65.194	0.118	-1.136
Calcium	0.122	-0.761	-0.002	76.993	0.117	-1.96
Phosphorus	0.122	-0.951	-0.001	57.76	0.119	0.071
PTH	0.126	2.123	0.002	131.132	0.123	2.991
Predialysis weight	0.111	-11.275	-0.018	97.066	0.101	-18.288

PAD

	HbA1c2		HbA1c3		HbA1c4	
	β_{11}	95% CI	B_{21}	95% CI	β_{11}	95% CI
Full Model	0.017	(-0.298, 0.331)	0.189	(-0.197, 0.576)	0.007	(-0.481, 0.496)

Variable Removed	β_{10}	HbA1c2 %change	B_{20}	HbA1c3 %change	B_{30}	HbA1c4 % change
Year of ESRD incidence	0.027	38.168	0.134	-40.94	-0.039	118.655
Census division	0.014	-17.979	0.19	0.441	0.01	25.465
Age	0.018	6.956	0.193	2.025	0.015	50.65
Sex	0.013	-32.341	0.182	-3.835	-0.002	422.089
Race	0.02	15.709	0.196	3.232	0.015	51.677
Ethnicity	0.02	17.117	0.193	2.041	0.01	30.849
Medicare/Medicaid dual eligibility	0.017	0.215	0.189	-0.032	0.007	-3.885
Median rent	0.017	2.796	0.189	0.084	0.007	2.933
Median household income	0.017	0.286	0.189	0.046	0.008	3.911
% living below poverty	0.018	7.142	0.189	-0.307	0.013	42.306
% unemployed	0.018	8.549	0.193	1.945	0.014	49.265
% with less than high school education_	0.001	-1157.14	0.18	-4.889	0.026	71.615
BMI	0.021	20.08	0.195	3.014	0.016	55.422
eGFR	0.012	-38.951	0.184	-2.554	-0.002	412.236
Heart failure	0.019	10.016	0.189	0.138	0.006	-21.39
Arrhythmias	0.006	-158.344	0.192	1.421	0.006	-19.43
Coronary artery disease	0.018	7.132	0.192	1.459	0.008	8.115
Other cardiac disease	0.013	-24.702	0.18	-5.006	0.003	-116.328
Peripheral arterial disease	0.037	54.839	0.165	-14.905	0.027	73.15
Hypertension	0.015	-9.74	0.193	1.757	0.008	9.514
Chronic obstructive pulmonary disease	0.019	13.363	0.192	1.454	0.008	7.232
Current tobacco use	0.016	-6.233	0.189	0.12	-0.006	228.63
Cancer	0.02	14.484	0.192	1.246	0.009	20.893
Alcohol dependence	0.016	-3.703	0.189	0.027	0.005	-45.493
Albumin	-0.004	487.396	0.196	3.256	0.073	90.078
nPCR	0.025	32.76	0.199	4.827	0.025	70.77
Hemoglobin	0.007	-126.436	0.176	-7.28	-0.007	205.657
Platelet count	0.016	-4.259	0.192	1.255	0.014	49.505
White blood cell count	0.01	-70.63	0.199	4.812	0.035	79.048
Ferritin	0.003	-497.783	0.185	-2.086	0.03	75.971
Mean arterial pressure	0.006	-171.737	0.165	-14.896	-0.051	114.105
Pulse pressure	0.019	11.3	0.191	1.131	0.01	28.713
Calcium	0.017	1.479	0.18	-5.065	-0.004	297.627
Phosphorus	-0.003	614.937	0.172	-10.188	-0.036	119.923
PTH	0.024	30.949	0.196	3.689	0.02	64.334
Predialysis weight	0.019	10.387	0.185	-2.019	-0.001	721.527

All-Cause Mortality

	HbA1c2		HbA1c3		HbA1c4	
	β_{11}	95% CI	B_{21}	95% CI	B_{31}	95% CI
Full Model	0.000	(-0.059, 0.060)	0.012	(-0.075, 0.098)	-0.001	(-0.099, 0.097)

Variable Removed	β_{10}	HbA1c2 %change	B_{30}	HbA1c3 %change	B_{30}	HbA1c4 %change
Year of ESRD incidence	0	-138.372	0.011	-7.983	-0.002	55.209
Census division	-0.001	139.272	0.009	-27.109	-0.005	82.099
Age	-0.017	102.426	-0.032	136.706	-0.091	98.973
Sex	-0.003	114.58	0.007	-56.043	-0.008	87.642
Race	-0.002	117.469	0.005	-113.293	-0.009	89.915
Ethnicity	-0.005	108.357	0.004	-163.369	-0.01	91.025
Medicare/Medicaid dual eligibility	0.001	22.348	0.012	0.077	-0.001	-31.78
Median rent	0.001	24.214	0.012	0.776	-0.001	34.203
Median household income	0	-34.868	0.012	-0.51	-0.001	-12.545
% living below poverty	0	-6.771	0.012	-0.052	-0.001	-6.143
% unemployed	0.001	31.553	0.012	1.382	-0.001	-50.242
% with less than high school education_	0	671.787	0.011	-5.909	-0.002	52.203
BMI	0	-44.876	0.01	-14.34	-0.001	27.393
eGFR	0.004	88.88	0.016	27.175	0.006	116.061
Heart failure	0.001	68.726	0.014	16.735	0.003	130.61
Arrhythmias	-0.002	127.116	0.01	-15.728	-0.004	75.891
Coronary artery disease	0.003	88.212	0.015	21.769	0.004	124.88
Other cardiac disease	-0.003	113.699	0.005	-132.428	-0.009	89.363
Peripheral arterial disease	0.003	87.062	0.013	11.489	0.002	146.464
Hypertension	0	-9.333	0.011	-1.767	-0.001	9.153
Chronic obstructive pulmonary disease	-0.001	140	0.007	-59.029	-0.007	85.732
Current tobacco use	0	-6.771	0.012	0.326	-0.001	13.691
Cancer	-0.004	110.47	0.005	-127.741	-0.005	82.065
Alcohol dependence	0	351.534	0.011	-4.566	-0.002	57.436
Albumin	-0.034	101.198	-0.005	325.271	0.029	103.196
nPCR	-0.012	103.368	-0.005	331.687	-0.017	94.647
Hemoglobin	-0.025	101.617	-0.026	143.946	-0.055	98.305
Platelet count	-0.005	107.815	0.006	-97.706	-0.004	78.423
White blood cell count	0.009	95.664	0.03	60.611	0.014	106.565
Ferritin	0.001	20.078	0.011	-1.236	-0.002	42.866
Mean arterial pressure	-0.007	106.189	0.001	-1955.3	-0.025	96.247
Pulse pressure	-0.001	138.246	0.002	-627.972	-0.018	94.956
Calcium	-0.002	119.468	0.007	-65.054	-0.008	88.049
Phosphorus	-0.001	154.449	0.01	-13.692	0.002	156.477
PTH	0.002	83.148	0.013	13.426	0.002	143.968
Predialysis weight	-0.016	102.577	-0.011	201.271	-0.025	96.267
